# Polarimetric biomarkers of peri-tumoral stroma can correlate with 5-year survival in patients with left-sided colorectal cancer

**DOI:** 10.1038/s41598-022-16178-3

**Published:** 2022-07-25

**Authors:** Jigar Lad, Stefano Serra, Fayez Quereshy, Mohammadali Khorasani, Alex Vitkin

**Affiliations:** 1 Department of Medical Biophysics, University of Toronto, Toronto, Canada; 2 Department of Laboratory Medicine and Pathobiology, University of Toronto, Toronto, Canada; 3 Department of Surgery, University of Toronto, Toronto, Canada; 4 Department of Surgery, University of British Columbia, Victoria, Canada; 5 Division of Biophysics and Bioimaging, Princess Margaret Cancer Centre, University Health Network, Toronto, Canada; 6 Department of Radiation Oncology, University of Toronto, Toronto, Canada

**Keywords:** Cancer, Biomarkers, Applied optics, Biophotonics, Imaging and sensing, Cancer imaging, Cancer microenvironment, Tumour biomarkers, Tumour heterogeneity, Oncology, Colorectal cancer, Prognosis, Pathology, Optics and photonics, Microscopy, Polarization microscopy, Transmission light microscopy

## Abstract

Using a novel variant of polarized light microscopy for high-contrast imaging and quantification of unstained histology slides, the current study assesses the prognostic potential of peri-tumoral collagenous stroma architecture in 32 human stage III colorectal cancer (CRC) patient samples. We analyze three distinct polarimetrically-derived images and their associated texture features, explore different unsupervised clustering algorithm models to group the data, and compare the resultant groupings with patient survival. The results demonstrate an appreciable total accuracy of ~ 78% with significant separation (p < 0.05) across all approaches for the binary classification of 5-year patient survival outcomes. Surviving patients preferentially belonged to Cluster 1 irrespective of model approach, suggesting similar stromal microstructural characteristics in this sub-population. The results suggest that polarimetrically-derived stromal biomarkers may possess prognostic value that could improve clinical management/treatment stratification in CRC patients.

## Introduction

Colorectal cancer (CRC) remains the second-leading cause of cancer-related deaths in North America^1–5^. Despite clinical advancements, minimal prognostication factors exist to allow improved personalization of the treatment. Currently, surgical resection is the only curative treatment for locoregional CRC, and clinicopathological characteristics (e.g., Tumour, Node, Metastases (TNM) staging) at presentation serve as the primary prognostication information for optimal cancer treatment selection strategies. In patients with lymph node metastasis (i.e., Stage III CRC), in addition to surgery, patients are considered for adjuvant systemic therapy in an attempt to improve survival. However, Stage III patients are heterogeneous, and differentiating prognostication tools to help avoid over- or under-treatment of patients are being actively sought out.

Most CRC prognostic biomarkers explored to date focus on genetic and epigenetic characteristics in the form of liquid biopsy^6–10^, with few now being considered/investigated for clinical workup for some sites (e.g., OncotypeDx, ColoPrint, ColoGuideEx, ColoGuidePro), for predicting distant recurrence in Stage II and III CRC^10^). However, multiple studies highlighting inadequate performance of most of these tests, along with their high costs^9,10^, drive the search for alternative sources of prognostic information. In this context, increasing evidence supports the prognostic value contained within the tumour micro-environment, such as tumour stromal architecture^11–15^, more specifically desmoplasia or the desmoplastic response (DR)^16–19^. DR is associated with the growth and structural remodeling of collagenous stroma in the most invasive tumour front regions. Assessing DR involves a three-class categorization of stromal maturity: immature, intermediate, and mature, where 5-year relapse-free survival (RFS) was found to correlate with stromal maturity^9–12^. Recent studies suggest DR as a RFS prognostic factor, independent of TNM staging and tumour grade^16–19^. However, despite its promise, assessing DR (either qualitatively or quantitatively) suffers from analysis subjectivity and inter-observer variability, making its clinical uptake difficult.

Second-harmonic generation has long been considered the gold standard microscopy technique for collagen imaging^20,21^. However, the high cost, long imaging times, and technical expertise/complexity associated with its femtosecond laser and advanced microscope components are not well suited for clinical deployment. Alternatively, comparatively simpler and more robust polarized light microscopy (PLM) has the potential to overcome these drawbacks while also offering high stromal (collagen) contrast and has indeed seen applications in various cancer tissue types such as breast, cervical, prostate, brain, and colon^22^. Further, recent studies have utilized the polarimetric Mueller matrix approach in colon cancer^23–26^ to characterize collagen structures by analyzing alterations in the various polarization properties of light as it interacts with tissue. However, these properties may not directly highlight the underlying structure and arrangement of collagen within the tissue sample, and thus much work has gone into interpreting and correlating the observed polarimetric parameter changes with their core biological or biophysical meaning. Yet with the advancement of artificial intelligence (AI) in recent years, an alternative to this detailed understanding/interpretation has emerged whereby researchers have used various machine and deep learning techniques to directly link up the rich biological information from the polarization properties with clinical diagnosis and prognosis^15,27–33^.

We have recently developed a simple and information-rich polarized light microscopy approach to characterize tumour stromal morphology. Technologically simpler than the Mueller matrix approaches, this methodology has enabled quantitative assessment of stromal maturity and the tumour-stroma ratio in breast cancer tissue samples; comparison against pathologist classifications yielded excellent initial agreement^34,35^. Another investigation correlated polarimetrically-derived stromal metrics with genetic prognostication results (OncoTypeDX), also with good agreements^36^. In the current work, we expand significantly on these initial studies by (1) increasing the polarimetric feature space for selecting potential prognostic biomarkers, (2) employing unsupervised clustering algorithms to analyze the data, and (3) correlating the results with actual clinical outcomes (5-year survival). The latter point is perhaps the most significant and potentially impactful, as the possible utility of the polarimetry method is gauged not against pathologist’s assessment nor against another prognostic biomarker score, but rather against actual patient outcomes. Working with unstained colorectal cancer histology slides, we show that a combination of novel polarized light imaging, texture-analysis and machine learning can stratify patients into survival-correlated groups, suggesting that tumour stroma may indeed contain information of significant prognostic value.

## Methods

### Ethics

Institutional ethics approval was obtained from participating hospital institutions (University Health Network in Toronto, Ontario, Canada). The need for patients’ consent was waived by the ethics board due to the retrospective nature of the study, anonymization of personal health information, and the results of H&E analysis already being discussed with patients. All procedures and handling of patient data were conducted in accordance with the University Health Network Research Ethics Board guidelines/approvals.

### Patient samples

This study included 32 archival surgical resection samples of patients with confirmed left-sided colorectal cancer. The inclusion criteria for patients were that they did not receive any neoadjuvant chemotherapy (i.e., prescribed prior to tumour removal during surgery) and were all categorized to have Stage III cancers after surgical intervention. All patients did receive adjuvant chemotherapy after surgery as is usually recommended in such cases to improve survival^3^. Clinical data (i.e., clinical outcomes—5-year survival) for these patients were available to assess correlations. For each case, a representative unstained slide with the deepest tumour invasion area was cut from the formalin-fixed and paraffin-embedded blocks to 4.5 μm thickness. Sample preparation involved chemical dewaxing to avoid possible polarization imaging artefacts^34–37^. No further processing was required for polarimetric imaging. An adjacent slide was cut, H&E-stained and imaged at 20 × magnification on an Aperio ScanScope CS (Leica Biosystems, USA) for the pathologists’ region-of-interest (ROI) selection.

### ROI selection and histology

**Figure 1:**
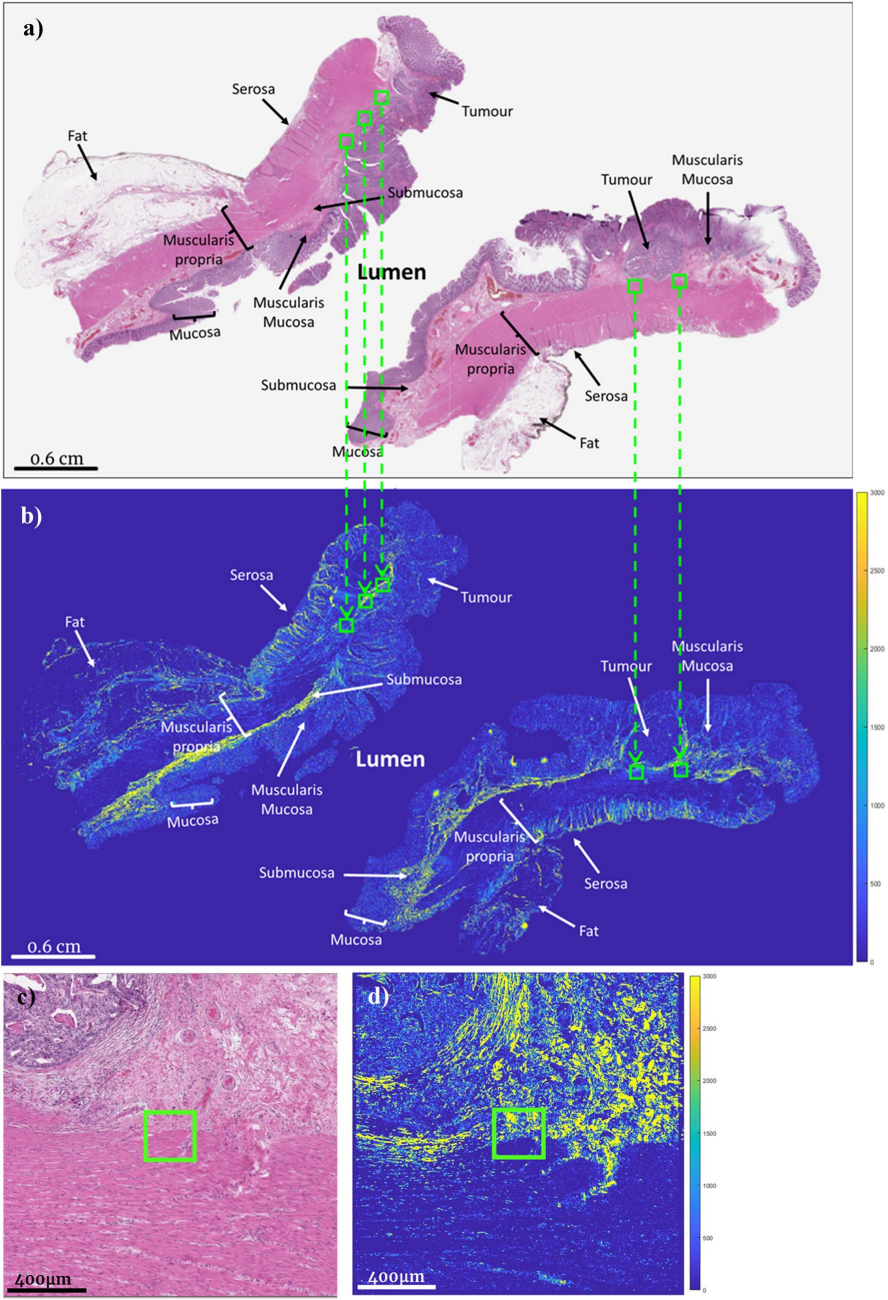
Whole-slide and ROI-based histologic and polarimetric imaging of a stage III CRC sample for qualitative ROI-based analysis. (**a**) H&E-stained slide and (**b**) corresponding polarimetric intensity image of an adjacent unstained slide (see *Polarimetric image quantification & analysis* section). (**c**) H&E and (**d**) polarimetric intensity zoomed-in images of the region around the right-most ROI in (**a**) and (**b**). The brighter areas visualize birefringent tissues that contain more collagenous stroma. The green squares represent the ROIs identified by the pathologist on the H&E images at the leading edges of the tumour, and the arrow show their transfer onto the polarimetric images. Images were obtained at 20 × magnification and were tiled and stitched for (**a**) and (**b**), and at 80 × for (**c**) and (**d**).

From the 32 adjacent H&E-stained slides, 297 regions of interest (ROIs, ~ 200 µm × 200 μm each) were selected by an experienced gastrointestinal pathologist (SS) in the regions along the invasive tumour front. The number of ROIs per patient slide depended on the size of the tumour and morphological characteristics of the stroma at the invasive tumour front, which was typically 6–10 (ranging from 3 (patient #5) to 14 (patient #8), refer to Fig. 3). The choice of ROI size was guided by previous studies, compromising between the need for robust statistics and capturing stromal heterogeneity while also maintaining reasonable spatial resolution/information^34,35,37^. The pathologist was blinded to the polarimetry images and clinical outcome data (i.e., 5-year survival) when choosing ROIs. These ROIs on the H&E images were then transferred onto the adjacent slides imaged polarimetrically (Fig. 1a,b), using visual tissue landmarks for guidance where necessary (Fig. 1c,d). Image processing and polarimetric analysis were then performed using MATLAB software (Mathworks, USA).

### Polarimetric method

**Figure 2:**
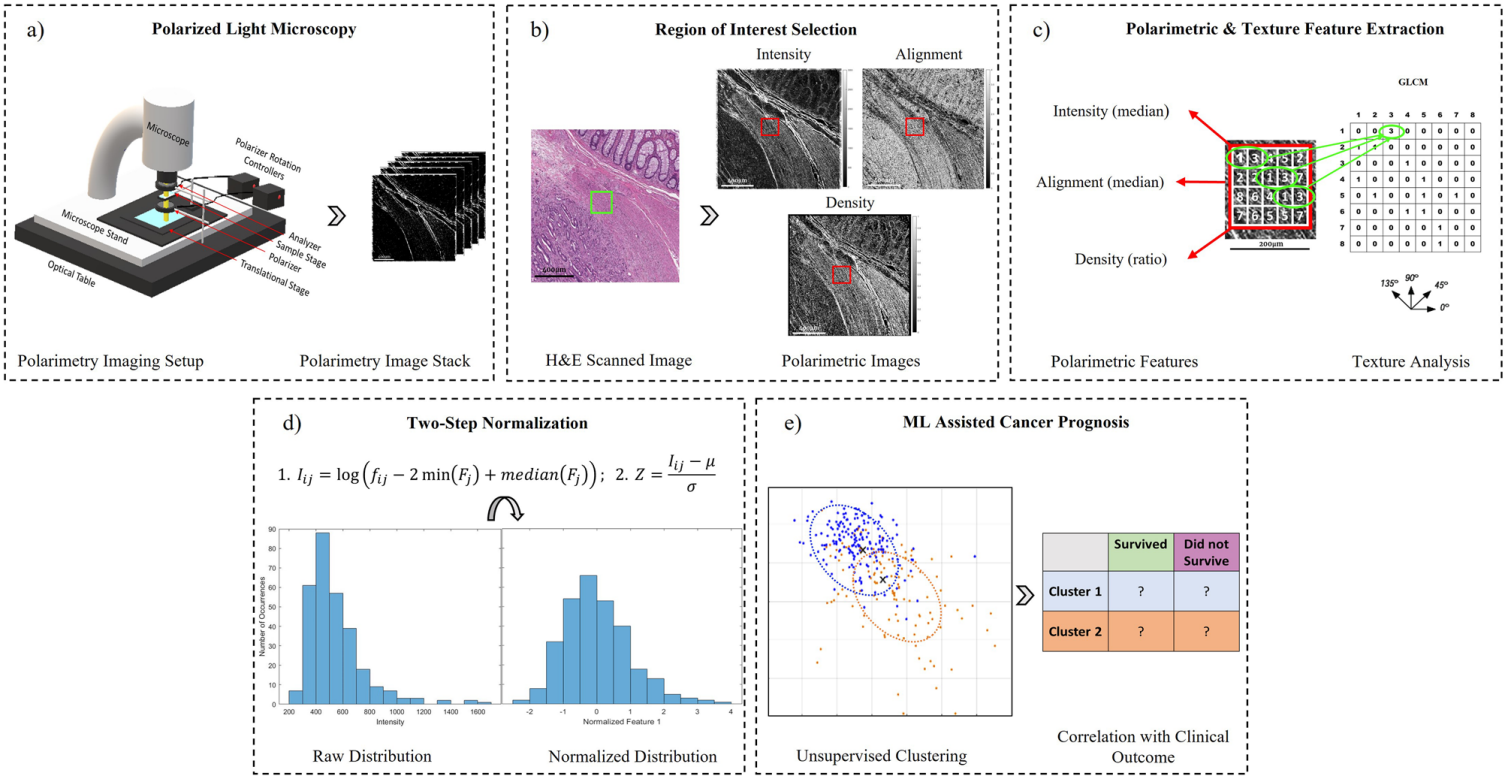
Workflow for proposed polarimetric imaging, analysis, and unsupervised clustering pipeline. (**a**) widefield polarimetric imaging of unstained tissue sample acquired at 18 angular orientations of the crossed linear polarizers (5° increments); (**b**) selection and calculation of novel polarimetric stromal features on the derived Intensity, Alignment and Density images from the 200 × 200 μm ROI identified by pathologist (green square); (**c**) calculation of median polarimetric values and GLCM texture features (contrast, correlation, energy, homogeneity and entropy) within ROI (red square), applied to each of the three derived polarimetric images; (**d**) two-step normalization to ensure feature values are zero-centered and resemble a normal distribution, as needed for clustering model input; (**e**) clustering of the ROIs using the polarimetric and texture features, with the resultant outputs clusters then assessed for correlations with patient outcomes.

Polarized light microscopy can enhance the contrast of birefringent materials (such as collagen) in a largely non-birefringent background of other tumoural tissue structures. No staining or contrast agent is required. Imaging for this study was done using our group’s novel polarimetric methodology previously described in detail^34–37^. Briefly, we used an AxioZoom V16 microscope (Zeiss, Germany) fitted with two linear polarizers (Thorlabs, LPVISE100-A) on computer controlled motorized rotation mounts (PRM1.MZ8, Thorlabs, USA). The pathology slides are positioned between the two linear polarizers oriented perpendicular (crossed) relative to each other. The crossed polarizer pair is then rotated though 90°, stopping in 5° increments, and at each angular stop a polarimetric image is taken (Fig. 2a). Analysis of the resultant 18-image stack enables several parametric tissue images to be derived that are independent of the measurement geometry; that is, we remove the artefactual image contrast variations that depend on the crossed polarizers’ orientation relative to the tissue’s birefringent collagenous structures. The three parametric images thus derived reflect (1) birefringence (collagen) signal intensity, (2) its 2D orientation in the transverse plane, and (3) its abundance (Fig. 2b). For this study, the ROIs were imaged at 80×, with an approximate field of view of 1.6 × 1.6 mm^2^ and resolution of 0.8 μm × 0.8 μm. Images were also acquired without the sample to enable a flood field correction.

### Polarimetric image quantification and analysis

Each of the polarimetric images produced—birefringence signal intensity, alignment, and abundance—has biophysical meaning, as previously described in greater detail^34,35^. Briefly, the measured light intensity varies significantly with the polarization measurement angle when sampling birefringent structures and does not modulate when other non-birefringent tissues are imaged (yielding a low dark signal as expected for crossed polarizers independent of their rotation angle).

1 $$MIR\left( {i,j} \right) \equiv \sqrt {\frac{{\mathop \sum \nolimits_{z = 1}^{Z} \left( {y_{{\left( {i,j,z} \right)}} - \overline{y}_{{\left( {i,j} \right)}} } \right)^{2} }}{z - 1}}$$

The intensity image (1) reflects the birefringence magnitude of the collagenous stroma, as quantified via the maximum intensity range (MIR, see Eq. (1)) or variation, at each pixel (*i*, *j*) across the 18 (Z) measurements. *y*_(*i*,*j*,*z*)_ represents a given pixel brightness at a specific angle (z), whereas $\overline{y}_{{\left( {i,j} \right)}}$ is the mean signal brightness for a particular pixel. For subsequent ROI analysis, the *median intensity value* within the region was calculated.

2 $$MAD = \frac{{\mathop \sum \nolimits_{i = 1}^{n - 1} \mathop \sum \nolimits_{j = i + 1}^{n} B\left( i \right) - B\left( j \right)}}{{\frac{{n\left( {n - 1} \right)}}{2}}}$$

The alignment image (2) reports the overall distribution of birefringent collagen fiber orientations. This is determined (see Eq. (2)) by noting the crossed-polarizers rotation angle at which the maximum signal intensity is produced at each pixel (B(*i*), B(*j*)). Then a 5 × 5 pixel sliding window (4 μm × 4 μm) is applied, where n is the number of pixels in the sliding window and a mean angular difference (MAD) between the pixels within the neighbourhood is calculated. Regions that have a low mean angular difference represent regions with more aligned structures (more common in healthy tissues), whereas the opposite is true for high mean angular differences (suggesting disorganized pathology region). The *median angular difference* was calculated for the ROI analysis.

3 $$R^{2} \equiv 1 - \frac{{\mathop \sum \nolimits_{z = 1}^{Z} \left( {y_{{\left( {i,j,z} \right)}} - f_{{\left( {i,j,z} \right)}} } \right)^{2} }}{{\mathop \sum \nolimits_{z = 1}^{Z} \left( {y_{{\left( {i,j,z} \right)}} - \overline{y}_{{\left( {i,j} \right)}} } \right)^{2} }}$$

Finally, the abundance image (3) is similar to the intensity but is amplitude-independent. That is, a highly birefringent collagen structure and a less birefringent one will yield different contrasts on the intensity image, yet both represent collagen. To derive such collagen-abundance image (R^2^, see Eq. (3)), angular modulation at each pixel is fitted to a theoretically derived sinusoid-squared dependence (*f*_(*i*,*j*,*z*)_)^35^, and the goodness-of-fit is noted. Then using a simple threshold of R^2^ > 0.75 (determined empirically through previous experiments), this approach can separate the collagenous stroma from other tissues. For the ROI analysis employed here, the *abundance metric* was reported as a ratio between the number of pixels above this threshold to the total number of pixels within the ROI.

### Texture analysis

To extract *additional* information from the three derived polarimetric images above, we turned to the commonly employed gray-level co-occurrence matrix (GLCM) approach and its associated texture features (Fig. 2c). These textural parameters represent second-order statistics acquired by analyzing the spatial relationships of neighbouring pixels^38^. The GLCM characterizes texture by calculating how often pairs of certain pixel values occur at a specified distance (typically one pixel to the nearest neighbour, or 0.8 μm corresponding to the optical resolution in our study) and direction (0°, 45°, 90°, 135°). The size of the GLCM is dependent on the number of gray levels, which can either encapsulate all pixel values present within the image or binned to specified range^38^. For this analysis, the GLCM was binned to 8 gray levels (1–8) to ensure a consistent size across all polarimetric images. A separate GLCM and its associated textural parameters were calculated for each angle, resulting in 4 gray-level matrices from which five textural features were derived and then averaged to provide direction-independent metrics. Here we calculated the five fundamental GLCM textural parameters of interest: *contrast*, *correlation*, *energy*, *homogeneity*, and *entropy*, as described by Haralick et al.^38^. A custom-built texture analysis program was written in MATLAB, taking advantage of the available functions whereby GLCM was applied directly to the ROIs. This analysis pipeline thus yielded 15 additional features for each ROI (5 texture features × 3 polarimetry images) to create a new feature space of size 18. These 18 features then served as the primary input to machine learning models used for this analysis.

### Machine learning

The introduction of machine learning in the form of unsupervised clustering models was pursued for two reasons: (1) due to the low patient numbers (n = 32), the scarcity of data may render even the simplest of supervised learning models (e.g., logistic regression) prone to overfitting, and (2) to solely assess the prognostic capabilities of the stromal features with no known labels and offer a quantitative approach for its evaluation. Prior to the polarimetric (i.e., median intensity, median alignment, and density ratio) and texture (contrast, correlation, energy, homogeneity, and energy averages) features serving as inputs to an unsupervised machine learning model, the 18 features for each ROI underwent pre-processing involving a two-step normalization comprised of a logarithmic transformation followed by Z-score normalization^39^ (Fig. 2d). This simple approach combines common methods for normalizing input data, otherwise inputs that are not normalized and are highly skewed can negatively affect model performance^40^.

There are various unsupervised approaches for clustering, to examine unlabelled data and find patterns or structures to maximize similarities of data points within a group (cluster) and minimize similarities between groups. For this study, we chose 3 promising clustering models: K-means, Fuzzy C-means, and Gaussian mixture models (GMM). K-means is an exclusive/deterministic/hard clustering method where data points can belong to only one cluster. The centre (centroid) is acquired by finding the mean value that has the minimal distance between the surrounding data points^41^. Fuzzy C-means is similar to K-means but employs overlapping/soft clustering where data points can belong to more than one cluster expressed in the form of weights or degrees of membership^41^. Similarly, its centroids are acquired by finding the mean value of the cluster. Gaussian mixture models are a form of probabilistic/soft clustering, which models the entire data set as a mixture of multiple Gaussian distributions which forms the cluster, with each point assigned a probability of belonging to either distribution^41^. Since we are working with a modest sized-data set (32 patients, ~ 300 ROIs) which limits the use of separate validation and test sets, the resultant clustering assignments of all three models were then directly compared to clinical outcomes, specifically to the 5-year survival status of the examined patients (Fig. 2e).

**Figure 3:**
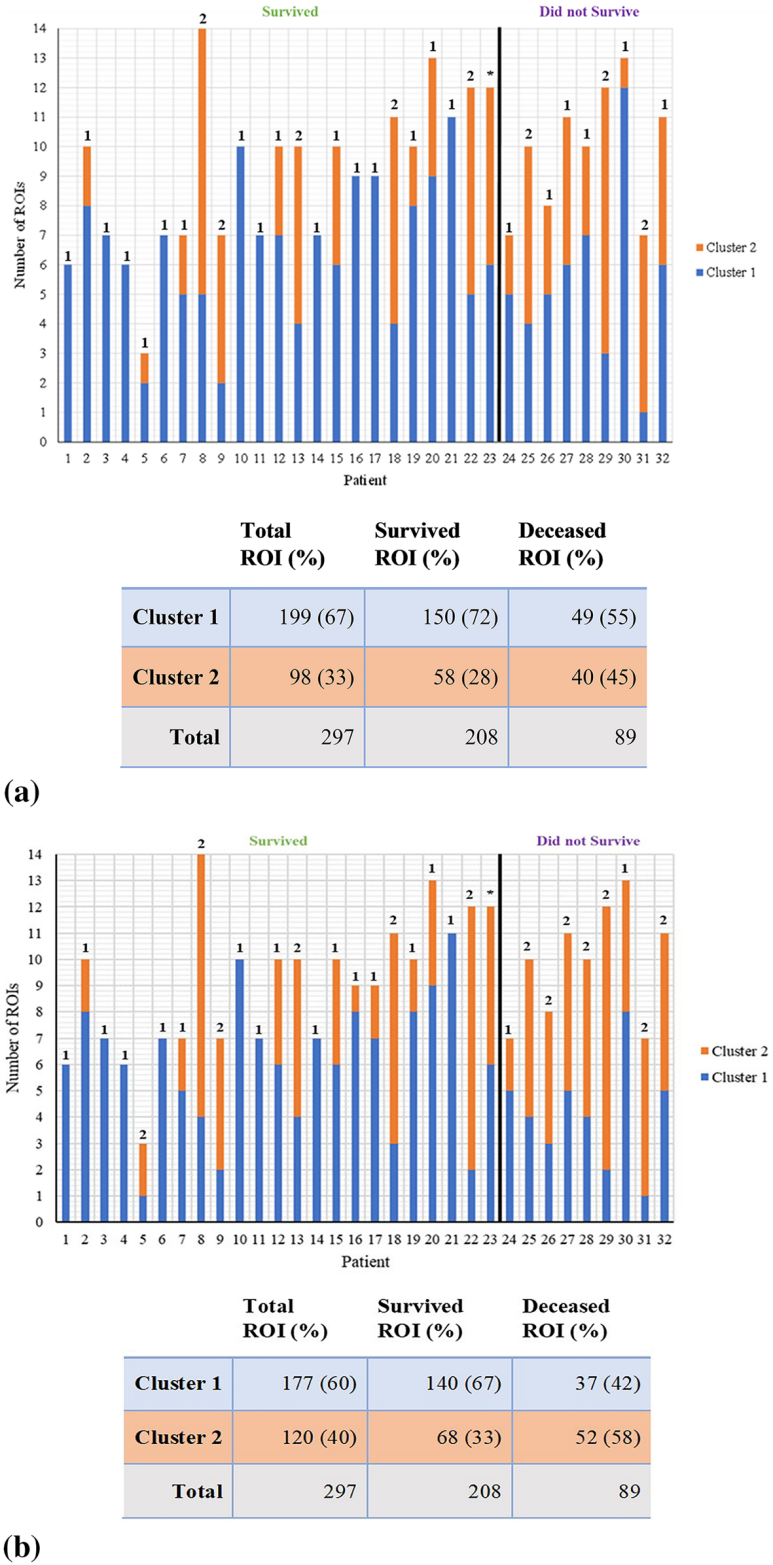

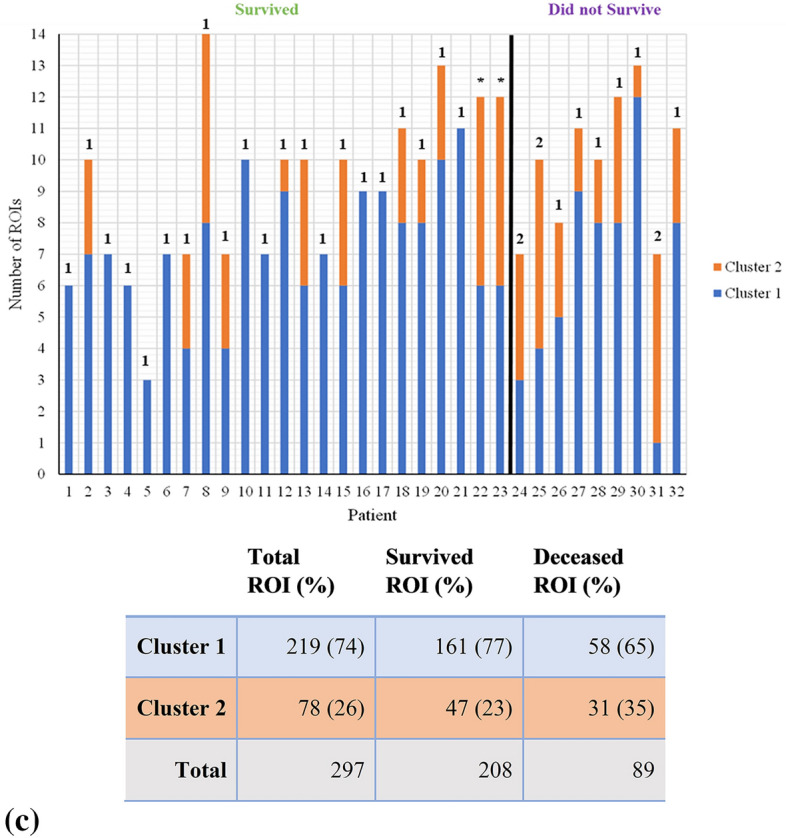
Resultant cluster assignments (blue = Cluster 1, orange = Cluster 2) of each region (n = 297) across all 32 patients for (**a**) K-means model, (**b**) Fuzzy C-means, (**c**) Gaussian Mixture Model. The black line partitions those who survived from those that did not. The number above each bar indicates the overall cluster assignment for that patient based on the majority-vote approach; the ambiguous “perfect tie” patients (*) remained unassigned (for details, see text). The table summarizes the assignment data for all 297 ROIs.

One methodological challenge was to then correlate clustering assignments of all 297 ROIs to each individual patient and thus his/her 5-year survival outcome. That is, the resultant labels for the ~ 10 ROIs from each patient (range 3–14) were mixed in many cases—some ROIs of a particular patient belong to Cluster 1, while others to Cluster 2. In order to determine where each individual patient belongs with their particular ROI classification pattern, we employed two different approaches: (1) majority-voting and (2) feature-averaging. For the former (1), if the ROIs cluster split was dominant one way or another (e.g., 70–30% or even 51–49%), the patient was assigned to that majority cluster. Two patients that happened to have an even number of ROIs *and* were evenly split (50–50%) could not be clustered this way (patients #22, #23—see Fig. 3). The alternative approach (2) averages the feature values (e.g., median intensity) across all ROIs selected for each patient. This overall patient-representing ROI is then assessed by inputting it back into the cluster-assigning algorithms. This ensures that all patients are assigned a cluster. For example, for the previously ambiguous unassigned patient #23 (having 6 ROIs belonging to Cluster 1 and 6 ROIs for Cluster 2), a simple average of the respective feature values across all ROIs (i.e., 12 ROIs × 18 features) yields a single feature vector (i.e., 1 averaged ROI × 18 averaged features) which then places this patient into either Cluster 1 or Cluster 2 (the former in this particular case). Using both the majority-vote and feature-averaging approaches, these resultant patient-representing clusters were then compared to clinical outcome (5-year survival) data.

### Statistical methods

Based on categorical data (patient survived *vs* did not survive, Cluster 1 vs. Cluster 2), a 2 × 2 contingency table was formed and assessed using the two-tailed Fisher’s exact test. The null hypothesis assumed no significant difference between the populations; a p value of < 0.05 indicated significant association between cluster groups and their corresponding 5-year survival outcomes. The sensitivity, specificity, positive predictive value, negative predictive value, and total accuracy were also determined to assess the prognostic capabilities of each of the three unsupervised models and the two methods for ROIs-to-patients assignments. The odds ratio (OR) was also calculated to assess the value added of the newly introduced GLCM texture features within our analysis.

## Results and discussion

We propose a polarimetry-based unsupervised clustering pipeline for categorizing the likelihood a patient is to survive after 5 years, using polarimetric imaging, image analysis and machine learning to achieve encouraging initial results. This expands our recently developed methodology^34–37^ by introducing GLCM texture features, implementing unsupervised machine learning clustering algorithms, and comparing our resultant cluster assignments directly with clinical data. This last point is particularly significant, as previous studies were largely correlated to pathology scoring and other (approximate and somewhat subjective) diagnostic/prognostic measures^34–37^. Comparing our predictions to actual patient survival outcomes is thus much more meaningful in the clinical context. Implementing machine learning enabled an efficient use of the 18 derived polarimetric biomarkers, allowing for a quantitative assessment of prognostic viability of collagenous stroma.

This study comprised 32 colorectal cancer patients all having stage III left-sided tumours with 23 (72%) 5-year survivors and 9 (28%) non-survivors. Given the asymmetry in the 5-year survivors *vs* non-survivor’s outcomes in the patient cohort and the variability in the numbers of ROIs per patient thus selected (Fig. 3a–c), many of the more popular supervised machine learning models (e.g., logistic regression, support vector machines) were deemed unsuitable. We thus proceeded with unsupervised clustering algorithms where such data asymmetries can be better tolerated (as the survival status is not part of the training process). Furthermore, despite supervised approaches being preferred for creating predictive models, unsupervised methods can directly assess whether any meaningful patterns exist within data without any known labels. In this study, we were blinded to the patient survival outcome, and only compared our clustering results with 5-year patient survival outcome *after* our polarimetric + machine learning analysis was concluded. Therefore, any significant correlations with survival outcome accentuates the inherent prognostic characteristics of collagen stromal features and suggests the significance of our polarimetric technique in extracting them quantitatively.

The clustering algorithms were trained on the individual ROIs using a parameter space of 18 features, grouping each ROI into one of two clusters. Figure 3 showcases the distribution of these ROIs across patients based on their survival status, for K-means, Fuzzy C-means, and Gaussian mixture model, respectively. As seen from all three panels in Fig. 3, majority of the individual regions belong to Cluster 1 as opposed to Cluster 2. GMM has the largest Cluster 1 population containing 219 (74%) ROIs compared to 199 (67%) and 177 (60%) for K-means and Fuzzy C-means, respectively. Conversely, the Cluster 2 is most populous in the Fuzzy C-means model (120 ROIs, or 40%) compared to K-means (98, 33%) and GMM (78, 26%). While there may be statistical and morphological reasons for the various similarities and differences in the clustering assignments of the three models, we now address the more interesting question of the correlation of these to the actual clinical survival on a patient-by-patient level.

**Figure 4:**
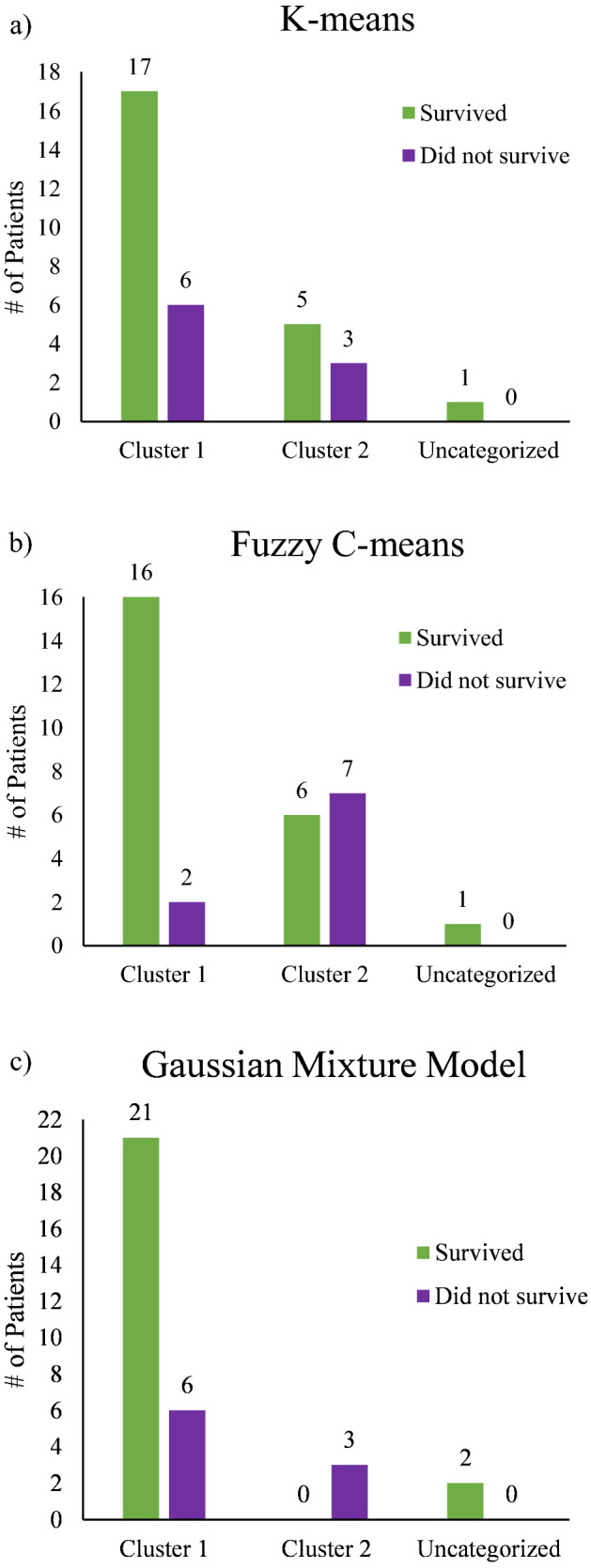
Cluster-Patient groupings (n = 32) using the majority-vote approach as determined by the three unsupervised clustering model. (**a**) K-means; (**b**) Fuzzy C-means; (**c**) Gaussian Mixture model. Green represents patients which survived after 5-years (n = 23) whereas purple denotes patients which did not (n = 9). Of note is the composition of Cluster 1 across all models, being primarily comprised of 5-year survivors (for details, see text).

**Figure 5:**
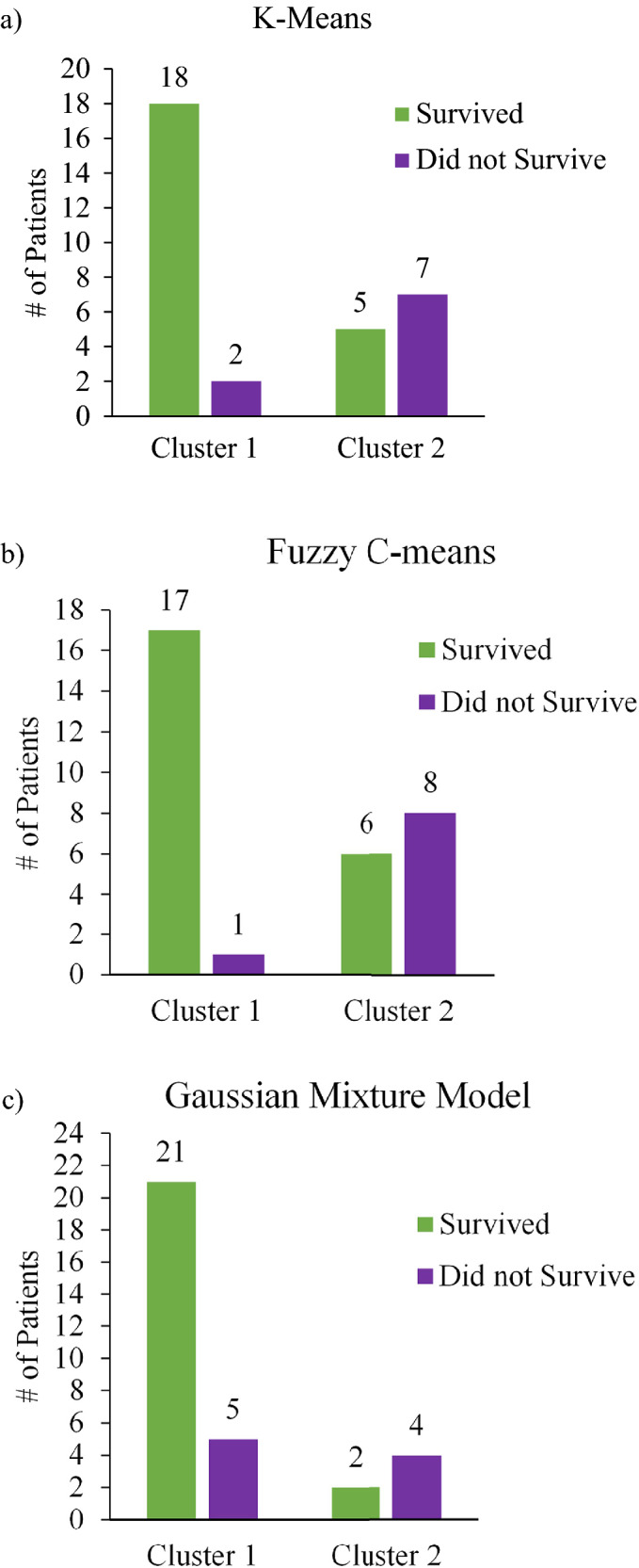
Cluster-Patient groupings (n = 32) using the features-averaged approach as determined by the three unsupervised clustering model. (**a**) K-means; (**b**) Fuzzy C-means; (**c**) Gaussian Mixture model. Green represents patients which survived after 5-years (n = 23) whereas purple denotes patients which did not (n = 9). Of note is the composition of Cluster 1 across all models, being primarily comprised of 5-year survivors (for details, see text).

Using the resultant ROI cluster labels from the three models as summarized in Fig. 3, we now present Fig. 4 (majority-vote) and Fig. 5 (feature-averaged) to examine the resultant patient-clustering clinical correlation with 5-year survival outcomes. Across all 3 algorithms, a similar distribution of patient outcomes was observed, supported by significant p-values (p < 0.05) (excluding for K-means/majority-vote), that is invariant of algorithm type or data handling approach. In terms of the majority-vote and features-average approaches, overall, the latter performed better on all metrics of analysis (Table 1), despite majority voting approach being perhaps more intuitive. As Figs. 4 and 5 illustrates, Cluster 1 primarily comprises of patients that survived at 5 years. In contrast, Cluster 2 does not contain such unequivocal composition; across all model types, Cluster 2 is ambiguous containing a similar number of patients that both survived and did not survive [excluding GMM/majority-vote, due to having its Cluster 2 being comprised of 100% non-survivors (Fig. 4c)].

**Table 1:** Summary of cluster model performances in relation to 5-year patient survival, expressed in the form of sensitivity, specificity, total accuracy, positive predictive value, negative predictive value, and p-values based on the two-tailed Fisher’s exact test.

Model	Sensitivity	Specificity	Total accuracy	Positive predictive value	Negative predictive value	P value (< 0.05)
K-means	0.3333	0.7727	0.7097	0.375	0.7391	0.6595
	0.7778	0.7826	0.7813	0.5833	0.9	0.0057
Fuzzy C-means	0.7778	0.7273	0.7419	0.5385	0.8889	0.0166
	0.8889	0.7727	0.7813	0.5714	0.9444	0.0037
GMM	0.3333	1.000	0.8000	1.000	0.7778	0.0207
	0.4444	0.913	0.7813	0.6667	0.8077	0.0385

The positive predictive value represents the population that did not survive and the negative predictive value for those that did survive. The top row for each model indicates results for the majority-vote approach performed on sample sizes of n = 31 for K-means, Fuzzy C-means, and n = 30 for GMM; the bottom row summarizes the performance of the features-averaged approach n = 32 for all three clustering models. Important to note that the respective tests are assuming the *ideal* groupings where Cluster 1 represents patients which survived, and Cluster 2 contains patients which do not. The results presented compare how well the findings reflect this assumption (for details, see text).

Ideally, the algorithms would have grouped all patients which survived into Cluster 1 and all those that did not into Cluster 2. This did not happen, as ~ 1/3 of the patients grouped into Cluster 2 (ranging from 3 to 14 across the six panels of Figs. 4 and 5, average = 9 patients) that contains roughly equal proportions of survivors and non-survivors. However, the situation for the other ~ 2/3 of the patients grouped into Cluster 1 (ranging from 18 to 27, average = 22 patients) is much more definitive, as this cohort is comprised primarily of survivors. Thus, the polarimetric approach, coupled with unsupervised machine learning clustering, can indeed offer novel prognostic information in 65–70% of the examined cases that were categorized into Cluster 1; based on our analysis, we can state with a high degree of certainty that these patients will be alive in 5 years. Conversely, for 30–35% of the examined patients, we cannot offer any new prognostic insights based on polarimetric analysis, as these patients got classified into a survival-ambiguous Cluster 2. This is a promising initial result, perhaps in the therapy selection process, to help separate ‘proceed-with-treatment’ cohort (Cluster 1) from ‘additional-prognostic-tests-required’ cohort (Cluster 2). This may assist in better resource allocation and in overtreatment avoidance (evidenced by high negative predictive values). But clearly further refinement of the methodology is needed to help define the optimal treatment trajectory of *all* examined stage III CRC patients, and not only ~ 70% as in the current study.

Out of all algorithm/approach combinations presented, the ones best suited for further refinement in future studies include Fuzzy C-means/majority-vote (Fig. 4b), K-means/features-average (Fig. 5a), and Fuzzy C-means/features-average (Fig. 5b). This is supported by relevant metrics such as specificity and negative predictive value that highlight the ability of our technique to stratify patients with positive prognosis. That is, the appreciable specificities of these models (78%, 72% and 77%) indicate the likelihood of our approach to correctly identify survivors at 5 years; more tellingly, the excellent negative predictive values (90%, 89% and 94%) highlight the accuracy of a ‘negative test result’ (belonging to Cluster 1) actually reflecting patients with favourable prognosis. Outside of the primary clinical prognostic marker (i.e., TNM sub-staging: IIIA, IIIB, IIIC), there is no widely accepted standard tool to differentiate CRC survival rates and assess prognosis in a reliable fashion. Attempted prognostication tools for this patient subgroup include OncotypeDx and ColoGuideEx. Specifically, Tian et al.^42^ assessed the overall accuracies of TNM (67.4%), OncotypeDx (67.4%), and ColoGuideEx (57.6%) for predicting survival in all stages of CRC; thus, our results appear to be competitive or slightly superior. That said, a more detailed comparison of specificities, sensitivities, and positive/negative predictive values will necessitate a larger patient cohort, as planned for our upcoming studies. However, the relatively high false positive cases (survivors in Cluster 2) of our technique are clearly problematic as indicated by specificities slightly lower than 80%; when paired with the low number of non-survivors, the resultant sensitivities (e.g., 89% in Fuzzy C-means/features-approach) and positive predictive values are suboptimal. A crucial area for improving this methodology will thus be the reduction of false positive rates, to make this technique valuable to not only ~ 2/3 of the examined patients but to their larger fraction.

Towards methodology improvement, it is important to understand what types of biomarkers are usefully contributing towards cluster separation, and perhaps stress those in future studies (for example, increase the number of GLCM texture features if suitable). We performed this analysis using the odds ratio (OR) approach on the results of Fig. 5, to evaluate the strength of association of each feature types (polarimetric and texture) with survival outcome, retrospectively (i.e., assuming the *ideal* groupings of Cluster 1 = survivors and Cluster 2 = non-survivors). As seen in Table 2, the addition of texture features leads to an overall improvement in cluster associations as opposed to the polarimetric features alone. The five second-order GLCM texture features drawn from each polarimetry image are thus extracting additional useful information. Despite the large uncertainty in the confidence interval ranges (attributable to the small dataset), this trend suggests further investigation to test if additional texture features (e.g., sum variance, difference of entropy) will allow for more specific information extraction.

**Table 2:** Contributions of the polarimetric and of the texture features to the cluster—survivor association, quantified via an odds ratio (OR), using the features-average approach.

Model	Polarimetry (95% CI)	Texture (95% CI)	Both (95% CI)
K-means	5.3 (1.0–25.2)	7.2 (1.3–31.5)	12.6 (2.0–67.4)
Fuzzy C-means	4.5 (0.8–20.7)	9.9 (1.7–52.7)	22.7 (2.6–262.3)
GMM	–	17.6 (1.8–13.5)	8.4 (1.3–49.3)

As seen, the use of both types of features seems to improve discrimination (note that for GMM, polarimetry-only OR could not be calculated due to zero survivors in Cluster 2 (results not shown)).

**Figure 6:**
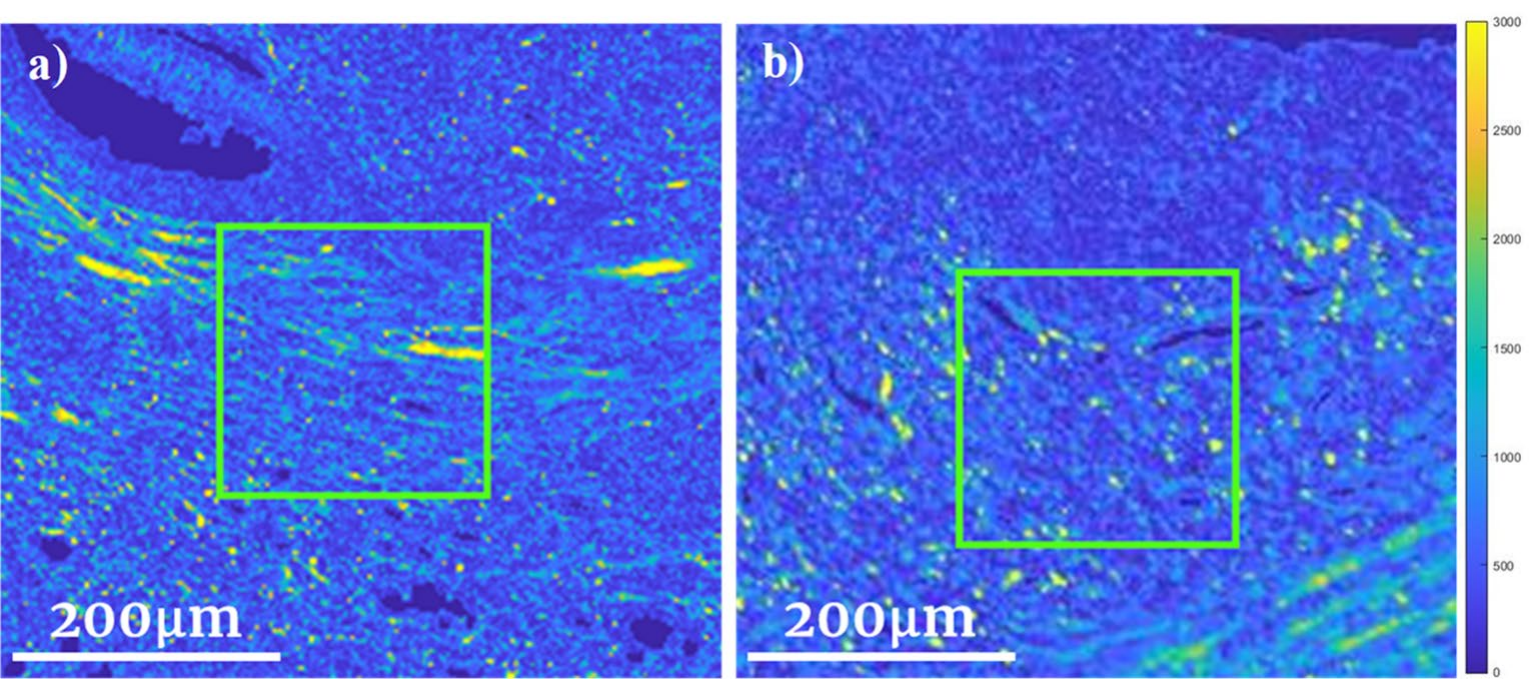
Two ROIs (green squares) on polarimetric intensity images of patient #30. (**a**) was deemed to belong to Cluster 1 with a weight of 90%, in comparison to (**b**) which was also considered to belong to Cluster 1, but with a weight of 56% (Fuzzy C-means algorithm). There are visible structural differences as is indeed reflected in the derived weights; this relative weight information could be used in future model refinements.

Moving forth, several avenues for methodology improvement appear promising. (1) Larger dataset—having a limited patient sample size meant that any slight variations in cluster distributions between algorithms or data handling approaches can alter the statistical significance of the observed correlations. (2) Supervised learning—related to above: as the patient sample size increases, the use of more popular supervised learning methods (e.g., logistic regression) that can also integrate relevant clinical features (e.g., age, sex, local T-stage, lymphovascular invasion, etc.) will become possible, to further assess whether our polarimetric analysis can yield independent and/or conjunctive prognostic biomarkers. (3) Additional GLCM features—the preliminary findings summarized in Table 2 suggest that the inclusion of more texture features (potentially as many as 20–25) may improve prognostication performance. (4) Cluster weighting—as depicted in Fig. 6, our current methods do not make use of the relative certainty (‘weight’) of an ROI’s specific cluster assignment; yet this is clearly valuable information reflecting underlying morphological differences that should be used in future models. (5) Automatic ROI selection—the varying number (e.g., patient #5 with 3 ROIs and patient #8 with 14 ROIs) and subjective choices of pathologist’s ROI selections likely affect the results and reduce robustness/objectivity of the approach. Implementing an automatic ROI selection model could address these shortcomings; however, unlike the other immediately-actionable improvement routes enumerated above, this represents a more formidable challenge that will likely require advanced AI/ML methodologies on its own^43,44^.

## Conclusion

Heterogeneity of Stage-III colorectal cancer is not currently reflected by the available prognostication tools, making personalization of treatment challenging for clinicians. One promising avenue to improve prognostic accuracy involves assessing stromal collagen in the TME, however objective quantifiable methods to do this are lacking. Here we present a simple, quantitative, and robust polarized light microscopy method for assessing tumour stromal features in 32 Stage III CRC patient samples. Supplementing the derived polarimetric images with second-order GLCM texture features and performing several unsupervised machine learning categorizations, we formed two clusters and then compared these with patient 5-year survival. A strong correlative association with survival was shown in 65–70% of examined patient cases. This demonstrates the useful information content present in PLM-measured CRC stromal features, but these promising initial results need to be considerably improved for potential use in the clinical setting. Several routes for such improvement—larger data sets, use of supervised learning, additional GLCM features, etc.—have been identified and will be pursued in future studies.

## Data Availability

Data underlying the results presented in this paper are not publicly available at this time but maybe obtained from the authors upon reasonable request.
